# Isolation, Identification and Phylogenetic Analysis of Canine Parvovirus Type 2c in Two Regions of Iran

**DOI:** 10.1002/vms3.70655

**Published:** 2025-10-24

**Authors:** Arshia Barzegar, Hadi Pourtaghi, Mohsen Lotfi, Mohammad Mahdi Ranjbar

**Affiliations:** ^1^ Department of Pathobiology, Ka. C. Islamic Azad University Karaj Iran; ^2^ Department of Microbiology, Ka. C. Islamic Azad University Karaj Iran; ^3^ Department of Quality Control Razi Vaccine and Serum Research Institute Agricultural Research, Education and Extension Organization (AREEO) Karaj Iran; ^4^ Department of FMD Razi Vaccine and Serum Research Institute Agricultural Research, Education and Extension Organization (AREEO) Karaj Iran

**Keywords:** canine parvovirus, genome characterization, isolation, phylogenetic analysis

## Abstract

**Background:**

Canine parvovirus Type 2 (CPV‐2) is a highly infectious canine virus that causes gastroenteritis in affected animals. As the virus emerged in the 1970s, it has undergone several mutations, resulting in three distinctive subtypes: CPV‐2a, CPV‐2b and CPV‐2c. The distinction between these subtypes is based on mutations in the *VP2* gene, which is the main structural protein of the virus.

**Objective:**

This study aimed to identify the circulating CPV‐2 strain in Iran through virus isolation and complete *VP2* gene sequencing and to determine its phylogenetic relationship with strains reported globally.

**Materials and Methods:**

Twenty‐five rectal swabs were collected from dogs displaying clinical symptoms of CPV‐2 infection. Samples were collected across the Alborz and Tehran Provinces of Iran during 2020 and 2021. Polymerase chain reaction (PCR) confirmed the presence of viral deoxyribonucleic acid (DNA) in the samples. The virus was isolated in the Crandell–Rees feline kidney (CRFK) cell line. The complete length of the *VP2* gene of the isolated virus was amplified using PCR via five primer pairs. PCR products were purified, Sanger sequenced and assembled in BioEdit software. A phylogenetic tree was constructed in MEGA11 software using the maximum likelihood method with the Tamura 3‐parameter model.

**Results:**

Glutamic acid at position 426 of the VP2 protein's amino acid sequence indicated that the isolate belonged to the CPV‐2c subtype. Phylogenetic analysis showed a close relationship between this isolate and isolates from Asia and Africa which are more recent than South America and Europe. Several mutations were detected in our isolate: A5G, F267Y, Y324I, Q370R and L583I, with the latter being a novel mutation never reported before. A5G and Q370R mutations have been detected only in isolates reported since 2016, suggesting that this study's isolate belongs to a newly appeared group of CPV‐2c viruses.

**Conclusion:**

This study identified the circulating CPV‐2 in Iran as the CPV‐2c variant, featuring a distinct L583I mutation. Further studies involving a more significant number of samples from across the country are required to assess the presence of this mutation in the circulating CPV‐2 population in Iran. Finding the possible impacts of this novel mutation on virus antigenicity is essential as it may affect the efficacy of CPV‐2 available vaccines.

## Graphical abstract

Canine parvovirus Type 2c (CPV‐2c) was isolated from infected dogs in Iran and identified through *VP2* gene sequencing. Phylogenetic analysis revealed close genetic links to Asian and African strains. A novel L583I mutation was detected, warranting further investigation into its impact on viral antigenicity and vaccine efficacy.

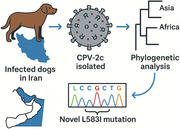

**Abbreviations**
BLAST: basic local alignment search tool
CPE: cytopathic effect
CPV‐2: canine parvovirus Type 2
CRFK: Crandell–Rees feline kidney
DMEM: Dulbecco's Modified Eagle Medium
DNA: deoxyribonucleic acid
FBS: foetal bovine serum
FPV: feline panleukopenia virus
MEV: mink enteritis virus
ORF: open reading frame
PCR: polymerase chain reaction
RNA: ribonucleic acid

## 1 Introduction

Canine parvovirus Type 2 (CPV‐2) is a significant pathogenic agent among domestic dogs and wild canines (Mazzaferro 2020; Steinel et al. 2001). This virus belongs to the family Parvoviridae, subfamily Parvovirinae, genus *Protoparvovirus* and together with feline panleukopenia virus (FPV) and mink enteritis virus (MEV), forms the species *Carnivore protoparvovirus*‐1 (Cotmore et al. 2019). CPV‐1, first identified in 1967, primarily affects young puppies and is considered less severe compared to CPV‐2, which emerged later as a highly contagious and more virulent strain. CPV‐2 infection results in haemorrhagic gastroenteritis and, in rare cases, acute myocarditis in neonates and young puppies (Voorhees et al. 2019; Kilian et al. 2018). The CPV‐2 genome consists of a linear, single‐stranded deoxyribonucleic acid (DNA) molecule approximately 5200 nucleotides in length, with two open reading frames (ORFs). The 3′ ORF encodes the non‐structural proteins NS‐1 and NS‐2, whereas the 5′ ORF encodes the structural proteins VP1 and VP2 (Reed et al. 1988; Parrish 1999). This DNA molecule is encapsidated within a non‐enveloped, icosahedral capsid approximately 25 nm in size, forming the virion. The capsid is composed of 6 copies of VP1 and 54 copies of VP2 (Tsao et al. 1991; Xie and Chapman 1996; Wu and Rossmann 1993).

CPV‐2 was first reported in 1978 (Appel et al. 1979), and phylogenetic studies suggest that it emerged in the early 1970s (Shackelton et al. 2005; Hoelzer et al. 2008). It is believed that the virus originated from a mutation in the FPV virus, which enabled it to bind to canine cells (Truyen 2006). Within a few months of the initial report, CPV‐2 spread among canines worldwide, reaching panzootic status (Parrish 1999). In 1980, a new strain of CPV‐2, named CPV‐2a, was identified. This strain replaced the original CPV‐2 globally by 1983 (Parrish et al. 1988). Another strain, CPV‐2b, was identified in the United States in 1984 (Parrish et al. 1991), and the most recent strain, CPV‐2c, was identified in Italy in 2000 (Buonavoglia et al. 2001).

The structural protein VP2 is the primary determinant of the virus's antigenicity, ability to bind to host cells and host range (Li et al. 2017). The CPV‐2a strain differs from the original CPV‐2 strain in amino acids at positions 87 (Met to Leu), 101 (Ile to Thr), 297 (Ser to Ala), 300 (Ala to Gly), 305 (Asp to Tyr) and 555 (Val to Ile), with the latter regressing to Ile in the CPV‐2b and CPV‐2c strains (Martella et al. 2006; Miranda and Thompson 2016). The original CPV‐2 and CPV‐2a strains possess Asn at position 426, whereas CPV‐2b has Asp and CPV‐2c has Glu at this position (Parrish et al. 1991; Buonavoglia et al. 2001). Consequently, the amino acid at position 426 of VP2 currently determines the antigenic strain of CPV‐2 (Zhou et al. 2017). Later studies detected a Ser to Ala substitution at position 297 in CPV‐2a and CPV‐2b subtypes. These newly discovered subtypes were named new CPV‐2a and new CPV‐2b, respectively (Martella et al. 2005).

According to recent studies, CPV‐2a accounts for approximately 45% of reported cases globally, followed by CPV‐2c at approximately 30% and CPV‐2b at approximately 22% (Hao et al. 2022). Previous studies in Iran reported CPV‐2a in 50% of cases, CPV‐2c in 32% and CPV‐2b in 18% (Ghajari et al. 2021). This study aimed to obtain the complete *VP2* gene sequence of CPV‐2, circulating in Iran to determine the phylogenetic relationship between the existing subtype in Iran and strains reported globally.

## 2 Materials and Methods

### 2.1 Sample Collection

Samples were collected from dogs exhibiting clinical signs consistent with parvoviral disease, including severe diarrhoea, vomiting, anorexia and lethargy. All dogs tested positive for CPV‐2 using a rapid antigenic test kit (flexy CPV Ag Test Kit, Ringbio, Beijing, China). A total of 25 samples were collected between 2020 and 2021 from 7 veterinary clinics: four in Tehran (16 samples) and 3 in Karaj (9 samples), located in Tehran and Alborz provinces, respectively. Tehran and Alborz are situated in the north‐central region of Iran. The geographical coordinates are as follows: Tehran—35.6892° N latitude, 51.3890° E longitude; Karaj—35.8559° N latitude, 50.9618° E longitude.

The samples were collected via rectal swabs using sterile techniques. The swabs were then placed in 3 mL of sterile phosphate‐buffered saline (PBS) and transported to the laboratory, where they were stored at −20°C until further analysis.

### 2.2 DNA Extraction and Detection of CPV‐2 in Samples

DNA extraction from the collected samples was performed using the DynaBio DNA extraction kit (Takapouzist Company), following the manufacturer's protocol. Detection of CPV‐2 DNA was achieved via polymerase chain reaction (PCR) using the 555_for_ (5′‐CAGGAAGATATCCAGAAGGA‐3′) and 555_rev_ (5′‐GGTGCTAGTTGATATGTAATAAACA‐3′) primer pairs, which amplified a 583‐base pair fragment. It utilized 2xTaq PCR Premix (Parstous), following the temperature protocol outlined by a previous study (Buonavoglia et al. 2001).

To confirm the accuracy of the PCR, the resulting products—including the sample, a positive control (HIPRADOG DP vaccine, HIPRA, Spain) and a negative control (sterilized distilled water)—were analysed via electrophoresis on a 1% agarose gel. The sample yielding the most evident band on a 1% agarose gel during electrophoresis was selected for further analysis.

### 2.3 Viral Isolation

The solution containing the rectal swab was first centrifuged at 1000 *g* for 10 min in an Eppendorf centrifuge to start viral isolation. The supernatant was collected and filtered through a 220‐nm filter (Millipore, USA). A 0.2‐mL aliquot of the filtered solution was supplemented with antibiotics—penicillin (1000 U), streptomycin (1 mg) and the antifungal amphotericin B (2.5 µg)—from Sigma‐Aldrich. For cell culture, the Crandell–Rees feline kidney (CRFK) cell line was used. One mL of cells was placed in a sterile 25‐cm^2^ culture flask. To this, 8 mL of Dulbecco's Modified Eagle Medium (DMEM) (Sigma‐Aldrich) supplemented with 1 mL foetal bovine serum (FBS) (Sigma‐Aldrich) was slowly added. The flasks were incubated at 37°C with 5% CO_2_ for 48 h, until the cells reached approximately 80% confluence. Subsequently, the prepared filtrate was added to the flask. Viral isolation was performed through three passages at 3‐day intervals. Upon observing cytopathic effects (CPEs), the cells were harvested by three cycles of freezing and thawing and stored at −80°C for further analysis.

### 2.4 *VP2* Gene Amplification

Following sample thawing, viral DNA was extracted using the DynaBio DNA extraction kit (Takapouzist). Five primer pairs were designed to amplify the full‐length *VP2* gene via PCR, as listed in Table 1. The primer design was based on the GenBank reference sequence M19296 and generated using OLIGO 7 software. PCR was conducted using 2xTaq PCR Premix (Parstous) following the temperature protocol, including 5 min initial denaturation at 95°C, followed by 35 cycles of 95°C for 35 s as denaturation, 60°C for 30 s as annealing and 72°C for 30 s as extension at 95°C. The PCR protocol finished by final extension at 72°C for 10 min.

**TABLE 1:** Nucleotide sequences of the designed primer pairs, their binding sites on the *VP2* gene and the corresponding lengths of the polymerase chain reaction (PCR) products.

Primer pair name	Forward primer sequence	Binding site (start–stop)	Reverse primer sequence	Binding site (start–stop)	PCR product length (bp)
1	5′‐AAAGAAGTAAACCACCACCTC‐3′	2694–2714	5′‐ACTAACTAAATGCAACTCACTC‐3′	3205–3184	512
2	5′‐TTGGTTGATGCAAATGCTTGG‐3′	3116–3136	5′‐AGTAGCTCCTTCAGATTGAG‐3′	3688–3669	573
3	5′‐GTAGACTAACACATACATGGC‐3′	3603–3623	5′‐GTTAATTCCTGTTTTACCTCC‐3′	4114–4094	512
4	5′‐GAAGATATCCAGAAGGAGATTG‐3′	4005–4026	5′‐TGGATTCCAAGTATGAGAGG‐3′	4426–4407	422
5	5′‐CAAATGAATATGATCCTGATGC‐3′	4305–4326	5′‐ATTTTTCTAGGTGCTAGTTG‐3′	4594–4575	290

### 2.5 Sequencing and Phylogenetic Analysis

The PCR products were gel‐purified and sequenced using the Sanger method on a genetic analyser (Applied Biosystems 3500, Thermo Fisher Scientific, US). The sequencing results were processed in BioEdit software V7.1 to assemble the full‐length *VP2* gene sequence, which spans 1755 base pairs, by aligning fragments and removing overlapping regions. This sequence has been registered in GenBank under accession number PP049248. Similar sequences available in GenBank were identified using the basic local alignment search tool (BLAST). All retrieved sequences, along with the sequence from this study, were aligned using the CLUSTAL W algorithm in Bioedit software (Thompson et al. 1994). Phylogenetic analysis and tree construction were performed in MEGA 10 software (Tamura et al. 2021), using the maximum likelihood method with the Kimura 3‐parameter model (Tamura 1992). The bootstrap consensus was based on 100 replicates and reflects the evolutionary history of the analysed isolates. Branch lengths indicate genetic distances between sequences. The best DNA/protein model for phylogenetic tree construction was selected using a model test program in MEGA 10.

## 3 Results

### 3.1 Detection of CPV‐2 Using Rapid Antigenic Test Kit and PCR

Among 25 samples that were collected from diarrhoeic dogs 17 cases were positive in a rapid antigenic test kit. Using 555 primer pairs which amplified a 583‐base pair fragment, 15 samples were detected as CPV‐2 positive.

### 3.2 Virus Isolation and *VP2* Gene Amplification

Among 15 positive samples, one sample that was related to a case that exhibited typic clinical signs of canine parvovirus disease and was positive in both rapid antigenic test, and PCR was chosen to virus isolation and to amplify the full‐length *VP2* gene. We could propagate this virus in CRFK cell line that demonstrated typic CPE through three passages.

### 3.3 *VP2* Gene Amplification

**FIGURE 1:**
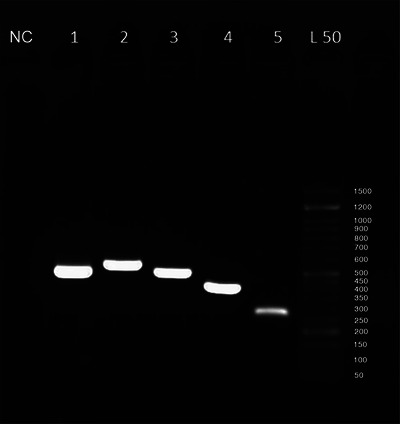
Agarose gel electrophoresis of five primer pairs used to amplify the complete length of *VP2* gene. L50 = 50 bp ladder; 1 = 512 nucleotides fragment from the first primer; 2 = 573 nucleotides fragment from the second primer; 3 = 512 nucleotides fragment from the third primer; 4 = 422 nucleotides fragment from the fourth primer; 5 = 290 nucleotides fragment from the fifth primer; NC = negative control.

All five primers produced clear bands during electrophoresis on a 1% agarose gel under UV light (Figure 1). This result demonstrates the effectiveness of the designed primers in successfully amplifying the entire *VP2* gene.

### 3.4 Sequencing and Phylogenetic Analysis

**FIGURE 2:**
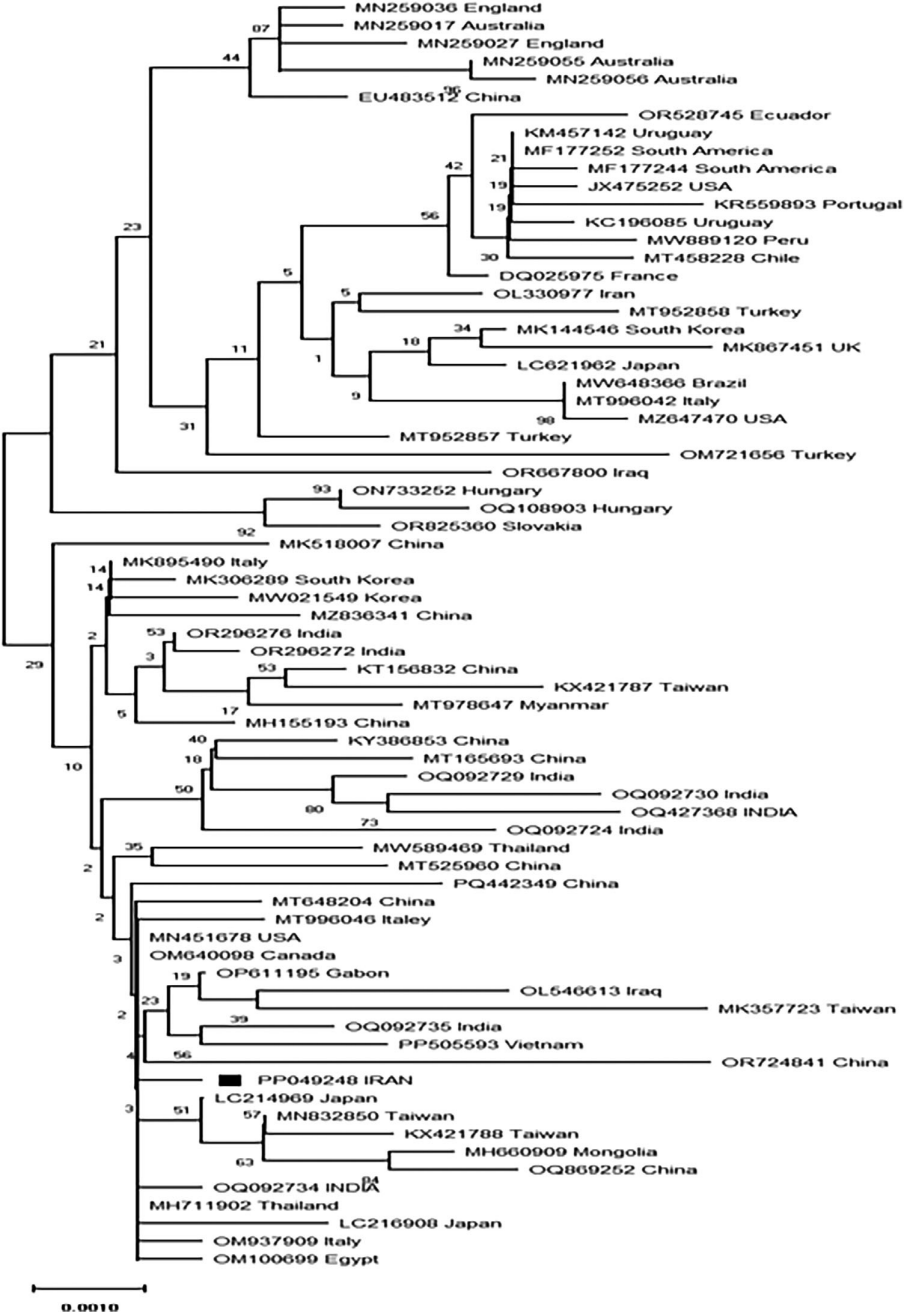
The evolutionary history was inferred using the neighbour‐joining method. The optimal tree with the sum of branch length = 0.10404453 is shown. The percentage of replicate trees in which the associated taxa clustered together in the bootstrap test (100 replicates) is shown next to the branches. The tree is drawn to scale, with branch lengths in the same units as those of the evolutionary distances used to infer the phylogenetic tree. The evolutionary distances were computed using the Kimura 2‐parameter method and are in the units of the number of base substitutions per site. The analysis involved 71 nucleotide sequences. Codon positions included were 1st + 2nd + 3rd + Noncoding. All positions containing gaps and missing data were eliminated. There were a total of 1751 positions in the final dataset. Evolutionary analyses were conducted in MEGA X.

Sequence alignment analysis showed five non‐silent mutations that leaded to five amino acid changes. The strain of the isolate was identified by comparing amino acid sequence with reference CPV‐2 strains available in GenBank. As shown in Table 2, the sample from this study has glutamic acid at position 426 of the VP2 protein, indicating it belongs to the CPV‐2c subtype. The phylogenetic tree of the viral isolate analysed in this study is shown in Figure 2.

**TABLE 2:** Amino acid positions in the VP2 protein of the isolate from this study (PP049248) that differ from those of the earliest reported isolates of each canine parvovirus (CPV) strain are listed in the table.

	VP2 amino acid positions														
Isolate name	Accession number	Subtype	5	87	101	267	297	300	305	324	370	375	426	555	583
USA78	M38245	CPV‐2	A	M	I	F	S	A	D	Y	Q	N	N	V	L
USA80	M24000	CPV‐2a	A	L	T	F	S	G	Y	Y	Q	D	N	I	L
USA84	M74849	CPV‐2b	A	L	T	F	S	G	Y	Y	Q	D	D	V	L
ITALY97	FJ005196	CPV‐2c	A	L	T	F	A	G	Y	Y	Q	D	E	V	L
IRAN21	PP049248	CPV‐2c	G	L	T	Y	A	G	Y	I	R	D	E	V	I

*Note*: The isolate names in the table are labelled according to the country and year of their identification.

## 4 Discussion

Since its emergence in the 1970s, CPV‐2 has been recognized as a significant canine infectious agent (Voorhees et al. 2019). The critical nature of parvoviral disease is underscored by global vaccination guidelines, which universally mandate the CPV‐2 vaccine for all dogs, irrespective of geographical location or circumstances (Day et al. 2016). Nevertheless, vaccination does not confer immunity in all cases, likely due to the existence of antigenically diverse viral strains (Decaro et al. 2020). Despite being a DNA virus, CPV‐2 exhibits a high nucleotide mutation rate comparable to that of ribonucleic acid (RNA) viruses (Shackelton et al. 2005). The rapid genomic evolution of CPV‐2 and its potential impact on the diminished efficacy of existing vaccines underscore the importance of sequencing and phylogenetic analysis of this virus (Bahoussi et al. 2022).

This study obtained the complete nucleotide sequence of the *VP2* gene from the isolated virus, consisting of 1755 nucleotides, determining this viral protein's corresponding amino acid sequence. This sequence has been deposited in GenBank under the accession number PP049248. A Glu residue at position 426 of the amino acid sequence identified the isolate as CPV‐2c. Moreover, several mutations were detected in the *VP2* gene of the virus that was isolated in this study compared to the first reported CPV‐2c isolate (FJ005196). The mutations A5G, F267Y, Y324I and Q370R observed in PP049248 were also identified in isolates retrieved from GenBank using BLAST and in studies reporting VP2 mutations (Sarabandi and Pourtaghi 2023). However, the L583I mutation was unique to this study's isolate among all examined sequences and according to large‐scale previous study, suggesting the emergence of a novel mutation in this isolate (Sarabandi and Pourtaghi 2023) To assess the presence of this mutation in the circulating CPV‐2 population in Iran, as well as its phylogenetic significance and origin, further studies involving a more significant number of samples from across the country are required. Additionally, molecular‐level investigations are necessary to evaluate the potential role of this mutation in factors such as alterations in viral virulence.

On the basis of the results obtained in phylogenetic tree, the sequence reported from Iran has CPV‐2c genotype and has the highest similarity to the sequences reported from East and Southeast Asia and also has some similarities to some sequences from Africa. The closest sequence similarity to the sequence reported from Iran (AC number: PP049248) is initially the Thailand sequence with accession number MH711902, then LC2149248 from Japan, OQ092734 and OM937909 from Italy, OM100699 from Egypt and finally OP611195 from Gabon. These sequences that belong to CPV‐2c along with some more divergent sequences from China, Mongolia, Vietnam and India are placed in the Clade. The Chinese sequence is more divergent and branches further away from the Iranian sequence in Clade A. Moreover, the CPV‐2c isolates analysed in the phylogenetic tree were categorized into two clades: Clade A and Clade B. Clade A comprises isolates reported exclusively from Asia and Africa. In contrast, Clade B includes isolates reported from South America and Europe. Notably, Clade A isolates are more recent than those in Clade B. Similarly, the short branch lengths observed for other Clade A members reflect this clade's relatively low genomic diversity (Umar et al. 2020). These findings suggest that PP049248 belongs to a relatively recent subgroup of CPV‐2c characterized by wide geographical distribution coupled with limited genetic diversity. A plausible explanation for this could be the high transmission rate of this CPV‐2c subgroup. Additionally, the existence of two distinct clades within the CPV‐2c lineage may reflect multiple independent pathways of global dissemination since the emergence of this strain. Besides, the previously reported sequence from Iran (accession number OL330977) belongs to the CPV‐2b genotype and forms a clade with sequences OR825360, MN259036, OM721656 and ON733252. The tree also contains other sequences related to the CPV‐2a genotype from the Americas and Europe, which are located on other branches and collectively form Clade D.

Earlier studies reported CPV‐2a as the predominant strain in Iran (Ghajari et al. 2021). However, more recent research indicates that CPV‐2c is becoming the dominant strain globally, including in regions where other strains previously predominated (Hao et al. 2022; Yip et al. 2020). The findings of this study reveal the presence of a mutated form of CPV‐2c within the canine population in Iran which contains A5G and Q370R mutations according to previous studies these mutations implicated in the increasing prevalence of CPV‐2c in Asia (Hao et al. 2022; Bahoussi et al. 2022). Moreover, the A5G and Q370R mutations are detected in isolates that were reported from 2016 onwards, which further supports the hypothesis that this study's isolate belongs to a recently formed subgroup of CPV‐2c (Bahoussi et al. 2022). These amino acids are not near any loops of VP2 protein, so the potential role of this mutant should be further evaluated.

## 5 Conclusion

The findings of this study suggest the presence of a mutated form of the CPV‐2c strain in two regions of Iran, which may indicate a potential emergence of this subtype as a new subtype in the country. However, further large‐scale studies are required to confirm this hypothesis and assess the impact of this mutation on the viral evolution and the efficacy of available vaccines.

Given the crucial role of the VP2 protein in the virus's pathogenicity, this mutation could have significant implications for vaccination strategies and disease control efforts, as it may influence viral virulence or alter the host range. However, it would be important to further investigate the subtypes present in the vaccines used in the region and correlate these with the findings. Future research should aim to explore the impact of these mutations and examine the effectiveness of existing vaccines in preventing CPV‐2 infections.

## Author Contributions

**Arshia Barzegar**: investigation, formal analysis, visualization, resources, writing – original draft preparation, validation (equal). **Hadi Pourtaghi**: conceptualization, methodology (lead), supervision (lead), project administration, writing – review and editing (equal), validation (equal). **Mohsen Lotfi**: methodology (supporting), writing – review and editing (equal), validation (equal). **Mohammad Mahdi Ranjbar**: methodology (supporting), supervision (supporting), review and editing (equal), validation (equal).

## Ethics Statement

The authors confirm that the ethical policies of the journal, as outlined on the journal's author guidelines page, have been adhered to. Approval from the appropriate internal ethics review committee has been obtained. This study was approved by the Iran National Ethics Committee on the Iran Islamic Azad University, Karaj, Research Ethics Committee (Code: IR.IAU.K.REC.1400.16).

## Consent

All authors have read and approved the final manuscript.

## Conflicts of Interest

The authors declare no conflicts of interest.

## Data Availability

The data supporting the findings of this study are available from the corresponding author upon reasonable request.
